# Human Retinal Organoid Modeling Defines Developmental Window and Therapeutic Vulnerabilities in MYCN-Amplified Retinoblastoma

**DOI:** 10.3390/ijms26178675

**Published:** 2025-09-05

**Authors:** Jinkyu Park, Gang Cui, Jiyun Hong, Han Jeong, Minseok Han, Min Seok Choi, Jeong Ah Lim, Sanguk Han, Christopher Seungkyu Lee, Min Kim, Sangwoo Kim, Junwon Lee, Suk Ho Byeon

**Affiliations:** 1 Department of Ophthalmology, Severance Eye Hospital, Institute of Vision Research, Yonsei University College of Medicine, Seoul 03722, Republic of Korea; aboutjins@naver.com (J.P.); chuikang617@gmail.com (G.C.); jnghan11@naver.com (H.J.); alstjr1599@naver.com (M.S.C.); lja8625@naver.com (J.A.L.); sangukhan29@gmail.com (S.H.); sklee219@yuhs.ac (C.S.L.); 2 Department of Biomedical Systems Informatics, Brain Korea 21 PLUS Project for Medical Science, Yonsei University College of Medicine, Seoul 03722, Republic of Korea; jyhong906@yuhs.ac (J.H.); swkim@yuhs.ac (S.K.); 3 Brain Korea 21 Project for Medical Science, Yonsei University, Seoul 03722, Republic of Korea; 4 Department of Medicine, Yonsei University College of Medicine, Seoul 03722, Republic of Korea; hanminseok1029@gmail.com; 5 Department of Ophthalmology, Institute of Vision Research, Gangnam Severance Hospital, Yonsei University College of Medicine, Seoul 06273, Republic of Korea; minkim76@yuhs.ac

**Keywords:** retinoblastoma, MYCN amplification, retinal organoids, tumorigenesis, retinal progenitor, precision medicine

## Abstract

MYCN amplification without concurrent RB1 mutations characterizes a rare yet highly aggressive subtype of retinoblastoma; however, its precise developmental origins and therapeutic vulnerabilities remain incompletely understood. Here, we modeled this subtype by lentiviral-mediated MYCN overexpression in human pluripotent stem cell-derived retinal organoids, revealing a discrete developmental window (days 70–120) during which retinal progenitors showed heightened susceptibility to transformation. Tumors arising in this period exhibited robust proliferation, expressed SOX2, and lacked CRX, consistent with origin from primitive retinal progenitors. MYCN-overexpressing organoids generated stable cell lines that reproducibly gave rise to MYCN-driven tumors when xenografted into immunodeficient mice. Transcriptomic profiling demonstrated that MYCN-overexpressing organoids closely recapitulated molecular features of patient-derived MYCN-amplified retinoblastomas, particularly through activation of MYC/E2F and mTORC1 signaling pathways. Pharmacological screening further identified distinct therapeutic vulnerabilities, demonstrating distinct subtype-specific sensitivity of MYCN-driven cells to transcriptional inhibitors (THZ1, Flavopiridol) and the cell-cycle inhibitor Volasertib, indicative of a unique oncogene-addicted state compared to RB1-deficient retinoblastoma cells. Collectively, our study elucidates the developmental and molecular mechanisms underpinning MYCN-driven retinoblastoma, establishes a robust and clinically relevant human retinal organoid platform, and highlights targeted transcriptional inhibition as a promising therapeutic approach for this aggressive pediatric cancer subtype.

## 1. Introduction

Retinoblastoma (RB) is a rare pediatric ocular malignancy primarily driven by biallelic inactivation of the RB1 tumor suppressor gene. However, a small subset of cases, accounting for approximately 1.5% of all retinoblastomas, is characterized by high-level amplification of the MYCN oncogene without accompanying RB1 mutations. These MYCN-amplified retinoblastomas exhibit distinct molecular signatures and demonstrate a clinically aggressive phenotype, characterized by rapid tumor growth, earlier age of onset typically within the first year of life, increased metastatic risk, and poor cellular differentiation [1].

MYCN, a member of the MYC family of transcription factors, is a well-established proto-oncogene implicated in regulating key cellular processes such as proliferation, differentiation, and apoptosis across multiple tissue types, and has been identified as a critical driver of tumorigenesis in various cancers [2]. During retinal development, progenitor cells undergo tightly regulated proliferation and differentiation processes, ultimately giving rise to mature, specialized retinal cell types. In this context, MYCN plays an essential role in maintaining the proliferative capacity of retinal progenitor cells [3]. However, MYCN overexpression disrupts this balance, leading to uncontrolled proliferation and impaired differentiation, characterized specifically by a failure of cells to exit the cell cycle and differentiate into mature retinal cell types [4]. Furthermore, MYCN overexpression profoundly perturbs critical molecular pathways and the retinal cellular microenvironment, promoting tumor initiation and progression—particularly in RB1-proficient retinoblastoma. Thus, a detailed investigation into how MYCN dysregulates developmental timing and disrupts regulatory networks in the developing human retina is crucial to better understand the early mechanisms underlying retinoblastoma tumorigenesis.

Experimental models have substantially advanced RB research but remain imperfect in recapitulating tumor initiation. Conventional cell lines (e.g., Y79, WERI-Rb1) offer reproducibility and utility for genetic and pharmacological studies [5], yet fail to reproduce the three-dimensional cytoarchitecture, cellular heterogeneity, and microenvironment of the developing retina [6]. In vivo approaches, including patient-derived xenografts (PDXs) and genetically engineered mouse models (GEMMs), provide valuable physiological contexts [7,8]. However, GEMMs often require additional genetic alterations beyond RB1 loss (e.g., p107 or p130 deficiency) to reliably induce retinoblastoma [9,10,11], raising concerns about their fidelity to human disease. PDXs, while preserving histological and genetic features [7,12], predominantly represent late-stage tumors and thus offer limited insight into tumor initiation. Moreover, species-specific differences further constrain direct translation to human retinoblastoma [13,14].

Recent advances in 3D retinal organoid technologies from human pluripotent stem cells (hPSCs) have provided a powerful platform that overcomes these limitations. Retinal organoids self-organize into laminated structures that faithfully recapitulate the cellular diversity, temporal dynamics, and spatial organization of the developing human retina [15,16,17,18], hereby enabling investigation of progenitor populations and developmental windows vulnerable to oncogenic transformation. As retinoblastoma originates during fetal retinal development [19], such models provide a particularly valuable platform for modeling both normal development and tumor initiation. Indeed, retinal organoid studies have reproduced RB tumorigenesis through RB1 inactivation [20,21] or MYCN overexpression [22]. However, key questions remain unresolved. Specifically, the exact developmental time window in which retinal progenitors are most vulnerable to MYCN-driven malignant transformation and the precise molecular mechanisms governing these processes require further clarification. Understanding how MYCN hijacks retinal developmental programs and interacts with diverse signaling pathways and transcriptional networks will be essential for the discovery of novel therapeutic targets and intervention strategies in retinoblastoma.

To elucidate the precise developmental context of MYCN-driven retinoblastoma, we utilized a human retinal organoid model to investigate how the timing of MYCN expression influences cellular susceptibility to oncogenic transformation. By overexpressing MYCN at distinct stages of retinal differentiation, we aimed to identify the developmental window of maximal vulnerability and characterize the associated molecular alterations. Through an integrated approach—including transcriptomic profiling, in vivo validation, and drug sensitivity screening—we sought to uncover critical oncogenic mechanisms and therapeutic vulnerabilities. Ultimately, this work establishes a robust platform for future mechanistic studies and the identification of targeted interventions in MYCN-driven retinoblastoma.

## 2. Results

### 2.1. MYCN Overexpression Drives Stage-Dependent Tumorigenesis and Maintains Undifferentiated Progenitor Characteristics in Retinal Organoids

**Figure 1:**
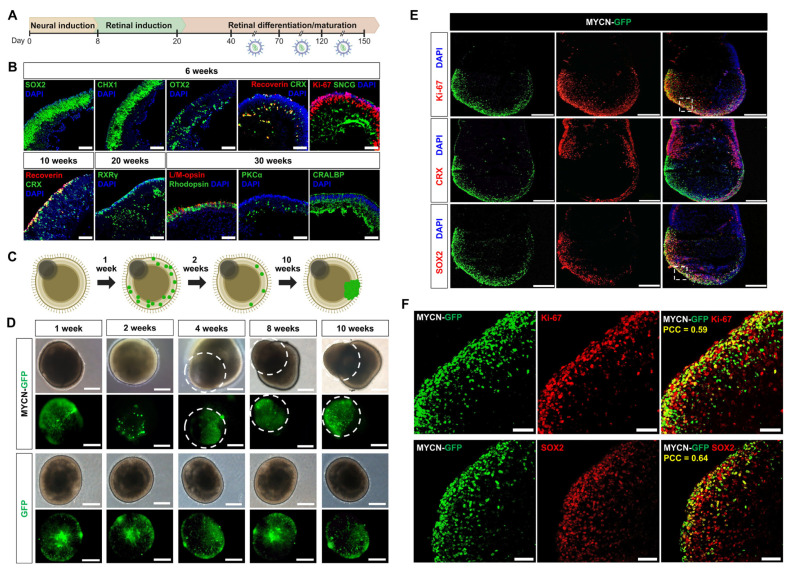
Tumorigenesis and characterization of MYCN-overexpressing tumors in human retinal organoids. (**A**) Experimental timeline depicting lentiviral transduction of MYCN-GFP at three distinct developmental windows (days 40–70, 70–120, and 120–150) of human retinal organoids. (**B**) Immunohistochemical characterization of retinal organoids at indicated differentiation stages (weeks 6, 10, 20, and 30). Organoids demonstrated proper retinal differentiation and maturation, evidenced by expression of markers for retinal progenitors (SOX2, CHX10), early photoreceptors (OTX2, CRX, Recoverin), retinal ganglion cells (SNCG), mature photoreceptors (Rhodopsin, L/M opsin), bipolar cells (PKCα), and Müller glia (CRALBP). Scale bars: 100 μm. (**C**) Schematic illustration summarizing tumorigenic progression following MYCN-GFP transduction. Numerous discrete focal GFP-positive cells are observed at 1-week post-transduction, but most of these cells disappear by week 2, leaving only a subset that retains GFP positivity. A fraction of these persistent cells subsequently expands to form tumors. The dark grayish structure at the upper left side of the spheroid represents the retinal pigment epithelium (RPE). (**D**) Representative time-lapse images showing morphological changes in retinal organoids following transduction with either MYCN-GFP or control GFP-only lentivirus. MYCN-GFP organoids exhibited focal and aggressive tumor growth (indicated by dashed circles), whereas GFP control organoids retained normal morphological characteristics. Scale bars: 200 μm. (**E**) Representative immunostaining images of MYCN-GFP tumor-bearing organoids showing robust cellular proliferation (Ki-67) and SOX2 expression, coupled with the absence of CRX expression, indicating an undifferentiated progenitor-like identity. Dashed boxes indicate regions shown at higher magnification. Scale bar: 100 μm. (**F**) High-magnification images confirming colocalization of MYCN-GFP with proliferation marker Ki-67 and progenitor marker SOX2. Pearson’s correlation coefficient (PCC) analysis revealed significant colocalization of MYCN-GFP with Ki-67 (PCC = 0.59) and SOX2 (PCC = 0.64), further supporting the progenitor identity of MYCN-induced tumor cells. Scale bar: 50 μm.

To determine if susceptibility to MYCN-driven tumorigenesis is dependent on the developmental stage of the human retina, we transduced retinal organoids with lentiviral vectors encoding either MYCN-GFP or a GFP-only control at three distinct windows: early (days 40–70), intermediate (days 70–120), and late (days 120–150) (Figure 1A). Normal retinal differentiation of organoids was verified by immunohistochemical confirmed through immunohistochemical analysis of stage-specific markers (Figure 1B).

Following successful transduction, both MYCN-GFP and GFP-control organoids initially exhibited comparable GFP expression. The organoids transduced with MYCN-GFP initially exhibited a marked reduction in GFP-positive cells by the second week, suggesting that MYCN overexpression triggered extensive apoptosis. Subsequently, a few surviving cells gave rise to focal, aggressively expanding neoplastic growths, characterized by disrupted organoid architecture. In contrast, control organoids transduced with only GFP retained their normal morphology and stable GFP expression throughout the observation period (Figure 1C,D).

The incidence of tumor formation was strongly dependent on the developmental stage at which MYCN was overexpressed. The highest frequency of tumorigenesis was observed in the intermediate stage (70–120 days), where 80% of organoids (24 of 30) developed tumors. This rate was significantly higher than that observed in both the early (25%; 5 of 20) and late (43.5%; 10 of 23) stages [χ^2^(2, N = 73) = 15.92, *p* < 0.001; Figure 1D].

To define the cellular identity of these neoplastic regions, we performed detailed immunofluorescence analysis. The tumor-like structures were composed of highly proliferative cells, as evidenced by robust Ki-67 expression. These cells uniformly expressed the retinal progenitor marker SOX2 but were notably devoid of the photoreceptor commitment marker CRX, indicating the persistence of an undifferentiated state (Figure 1E). To quantify these spatial relationships, we performed co-localization analysis, which confirmed a significant positive correlation between the MYCN-GFP signal and both Ki-67 (PCC = 0.59) and SOX2 (PCC = 0.64) (Figure 1F). Conversely, there was a negligible correlation with CRX (PCC = 0.02) (Figure 1E).

Collectively, these findings demonstrate that retinal progenitors have a peak window of susceptibility to MYCN-driven transformation between 70 and 120 days. The data suggest that MYCN promotes tumorigenesis by maintaining cells in a proliferative, undifferentiated progenitor-like state while preventing their differentiation into the photoreceptor lineage. Based on these features, we designate these transformed models as MYCN overexpressing retinoblastoma organoids (MYCN^O/E^-RBOs).

### 2.2. Organoid-Derived MYCN^O/E^-Cells Are Functionally Tumorigenic and Resemble Human Disease In Vivo

**Figure 2:**
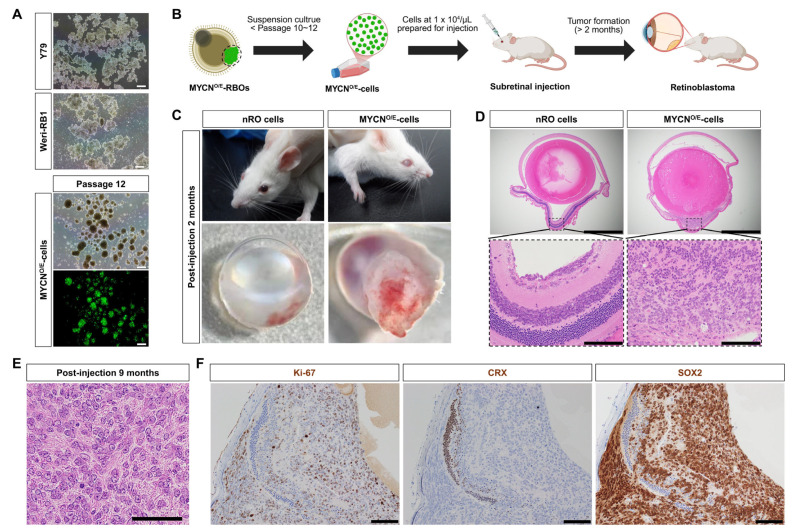
Establishment and in vivo tumorigenicity of MYCN-overexpressing cell lines derived from retinal organoids (MYCN^O/E^-cells). (**A**) MYCN^O/E^-cell lines derived from retinal organoid tumors were cultured under suspension conditions and underwent single-cell dissociation at each passage. Dissociated cells consistently formed tumor spheres over multiple passages, exhibiting morphology similar to established human retinoblastoma cell lines (e.g., Y79, WERI-Rb1). Scale bar: 100 μm. (**B**) Schematic illustration of the subretinal xenograft procedure performed using MYCN^O/E^-cells. (**C**) Representative images of immunodeficient mice two months post-subretinal injection of MYCN^O/E^-cells. Injected eyes exhibited clinical leukocoria and significant intraocular tumor formation, while control eyes injected with normal retinal organoid (nRO) remained morphologically normal. (**D**,**E**) Histopathological characterization of xenograft tumors at 2 months (**D**) and 9 months (**E**) post-injection revealed highly aggressive and poorly differentiated tumors, composed of densely packed, pleomorphic, hyperchromatic cells with enlarged nuclei, prominent nucleoli, and high nuclear-to-cytoplasmic ratios, closely recapitulating clinical MYCN-amplified retinoblastoma. Rosette structures were notably absent. Scale bar: 100 μm (**D**); 50 μm (**E**). (**F**) Immunohistochemical analyses of xenograft tumors at 9 months post-injection demonstrated robust proliferation (Ki-67 positivity), strong retinal progenitor identity (SOX2 positivity), and lack of photoreceptor differentiation (absence of CRX expression), further confirming the undifferentiated state of tumors. Scale bars: 100 μm.

To evaluate the tumorigenic potential and cellular properties of MYCN-overexpressing cells (MYCN^O/E^-cells), GFP-positive tumor regions were isolated from MYCN^O/E^-RBOs and subsequently cultured in vitro. Initially, isolated GFP-positive cells showed limited proliferative capacity under adherent culture conditions. To enhance their growth, we shifted to suspension cultures, a standard technique utilized for retinoblastoma cell lines such as Y79 and WERI-Rb1. In suspension, MYCN^O/E^-cells dissociated into single cells formed homogeneous tumor spheres that proliferated consistently over at least 12 passages, exhibiting morphological characteristics similar to established human retinoblastoma cell lines (Figure 2A).

To confirm the in vivo tumorigenic capability of MYCN^O/E^-cells, we performed orthotopic subretinal injections into immunodeficient NOD-SCID mice (Figure 2B). Two months post-injection, mice developed prominent intraocular tumors resembling leukocoria, while control mice injected with normal retinal organoid (nRO) exhibited no morphological abnormalities (Figure 2C). Histopathological analysis at this stage revealed marked disruption of normal retinal architecture, accompanied by the loss of retinal layer integrity (Figure 2D).

We further assessed the long-term tumorigenic potential by examining mice nine months post-injection. At this stage, MYCN^O/E^-cells gave rise to aggressively expanding intraocular tumors composed of densely packed, small undifferentiated cells with hyperchromatic nuclei, prominent nucleoli, and a high nuclear-to-cytoplasmic ratio. The tumor cells displayed marked nuclear enlargement and pleomorphism, closely resembling the hallmark pathological features of clinical MYCN-amplified retinoblastoma. Notably, the characteristic rosette structures typically observed in classical retinoblastoma were absent, underscoring the highly undifferentiated nature of these tumors (Figure 2E).

Immunohistochemical analyses further characterized these tumors, revealing robust proliferative activity (Ki-67 positivity) and prominent SOX2 expression, indicative of an undifferentiated, progenitor-like identity. Consistent with impaired photoreceptor differentiation observed in MYCN-driven tumors, CRX expression was undetectable within these tumor tissues (Figure 2F).

Collectively, these findings demonstrate that MYCN-overexpressing retinoblastoma organoids generate stable cell lines capable of robust tumor formation in vivo, closely resembling the key histopathological and molecular features of human MYCN-amplified retinoblastoma. Thus, our retinal organoid-derived MYCN-overexpressing models—including organoids, stable cell lines, and xenografts—represent clinically relevant disease models for investigating and developing novel therapeutic strategies.

### 2.3. Transcriptomic Analysis Identifies Distinct Molecular Signatures in MYCN-Overexpressing Retinoblastoma Organoids

**Figure 3:**
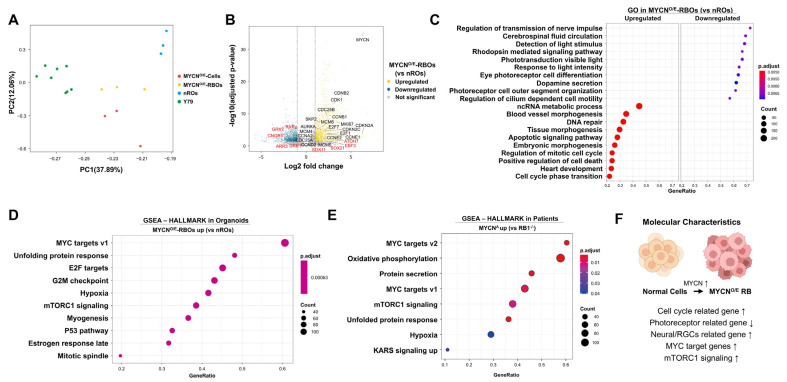
Transcriptomic profiling reveals distinct molecular signatures and oncogenic pathways in MYCN-overexpressing retinoblastoma organoids (MYCN^O/E^-RBOs) and similarity to patient-derived tumors. (**A**) Principal component analysis (PCA) comparing transcriptomic profiles among MYCN-overexpressing retinal organoids (MYCN^O/E^-RBOs), MYCN^O/E^-RBOs derived cell lines (MYCN^O/E^-cells), normal retinal organoids (nROs), and Y79 retinoblastoma cells (RB1^-/-^/MYCN^A^). MYCN^O/E^ samples formed distinct clusters separate from nROs and Y79, indicating unique transcriptional programs driven by MYCN overexpression. (**B**) Volcano plot showing significantly upregulated (1533 genes, yellow) and downregulated (892 genes, blue) differentially expressed genes (DEGs) between MYCN^O/E^-RBOs and nROs (adjusted *p* < 0.05, |Log2FC| > 1). (**C**) Gene Ontology (GO) enrichment analysis highlighting significantly downregulated biological processes (photoreceptor differentiation, phototransduction, sensory perception of light stimulus) and significantly upregulated biological processes (cell cycle phase transitions, mitotic cell cycle regulation, DNA repair pathways) in MYCN^O/E^-RBOs compared to nROs. (**D**,**E**) Gene Set Enrichment Analysis (GSEA) demonstrated significant enrichment of MYC target genes, unfolded protein response, mTORC1 signaling, and hypoxia-related pathways in MYCN^O/E^-RBOs (**D**) and independent MYCN-amplified patient tumor datasets (**E**). Concordant pathway activation between MYCN^O/E^-RBOs and patient-derived tumors supports the clinical relevance of this organoid model. (**F**) Schematic summary highlighting key molecular signatures identified from transcriptomic analyses. MYCN overexpression in retinoblastoma drives increased expression of MYC target genes, enhanced mTORC1 signaling, activation of cell-cycle-related and neural/retinal ganglion cell (RGC)-related genes, and downregulation of photoreceptor differentiation-related genes, collectively promoting a proliferative, undifferentiated retinal progenitor-like phenotype.

To comprehensively define the molecular characteristics of MYCN-driven tumorigenesis in human retinal organoids, we performed transcriptomic profiling and compared MYCN-overexpressing retinoblastoma organoids (MYCN^O/E^-RBOs), MYCN^O/E^-RBOs derived cell lines (MYCN^O/E^-cells), normal retinal organoids (nROs), and the *RB1*-deficient retinoblastoma cell line Y79. Principal Component Analysis (PCA) revealed clear transcriptional distinctions between MYCN-overexpressing samples (MYCN^O/E^-RBOs and MYCN^O/E^-cells) and controls (nROs and Y79 cells), indicating that MYCN overexpression drives a unique transcriptional program distinct from both normal retinal development and RB1-deficient retinoblastoma (Figure 3A).

Differential expression analysis comparing MYCN^O/E^-RBOs and nROs identified a total of 2445 significantly dysregulated genes (1533 upregulated, 892 downregulated; adjusted *p* < 0.05, |Log_2FC| > 1) (Figure 3B, Supplementary Data S1). Genes prominently upregulated included those involved in cell cycle progression and proliferation (e.g., MKI67, CCNA2, CCNB2, CCNB1, CCND2, CCNE1, CCNE2, CDKN2A, CDKN2C, CDK1, E2F1, E2F7, MCM4-6, SKP2, CDC25A/B, AURKA) and neural or retinal ganglion cell (RGC)-associated transcription factors (SOX11, ATOH7, EBF3, SOX21). Conversely, genes critical for cone photoreceptor function (e.g., ARR3, RXRγ) and phototransduction processes (CNGB3, GRK7, GRK1) were significantly downregulated, reflecting impaired photoreceptor differentiation.

Gene Ontology (GO) analysis further highlighted pronounced differences in biological processes between MYCN^O/E^-RBOs and nROs (Figure 3C, Supplementary Data S2). Processes significantly downregulated in MYCN^O/E^-RBOs included photoreceptor cell differentiation, rhodopsin-mediated signaling, phototransduction, and sensory perception of light stimuli, as well as cilium-dependent cell motility and outer segment organization. Conversely, upregulated processes predominantly involved cell cycle regulation, mitotic cell cycle transitions, and DNA repair mechanisms, consistent with enhanced proliferation.

To further validate our retinal organoid model as a representative platform for studying MYCN-driven retinoblastoma, we performed Gene Set Enrichment Analysis (GSEA) comparing MYCN^O/E^-RBOs with nROs and independently analyzed publicly available datasets from MYCN-amplified patient retinoblastomas relative to RB1-deficient tumors. GSEA revealed highly consistent pathway enrichment between MYCN^O/E^-RBOs and patient-derived MYCN-amplified samples. Both groups demonstrated significant enrichment in hallmark pathways associated with MYC targets (v1 and v2), unfolded protein response, mTORC1 signaling, and hypoxia-related pathways (Figure 3D,E, Supplementary Data S3 and S4). The strong concordance between our retinal organoid model and clinical MYCN-amplified retinoblastoma further supports the validity and clinical relevance of our organoid system for investigating MYCN-driven tumor biology.

A concise visual summary integrating these transcriptomic insights illustrates the key molecular characteristics of MYCN-driven retinoblastoma: activation of MYC/E2F transcriptional networks, enhanced proliferation and mTORC1 signaling, increased neural/RGC-associated transcription factors, and diminished expression of photoreceptor-specific genes, collectively reflecting a shift toward a proliferative, undifferentiated retinal progenitor-like state (Figure 3F).

### 2.4. MYCN-Overexpressing Retinoblastoma Cells Exhibit Selective Sensitivity to MYC-Targeted Therapeutic Agents

To identify therapeutic vulnerabilities specific to MYCN-driven retinoblastoma, we assessed drug sensitivity profiles of MYCN-overexpressing cells (MYCN^O/E^-cells) derived from retinal organoids compared with the established retinoblastoma cell line Y79 (RB1^−/−^, MYCN-amplified). Cell viability assays (WST-1 assay) were conducted using compounds categorized according to their mechanism of MYC inhibition: transcriptional inhibitors (THZ1, Flavopiridol, Roscovitine, JQ1, I-BET), translational inhibitors (Dactolisib, Rapamycin), and regulators of MYC protein stability (Alisertib, Volasertib, Prexasertib).

**Figure 4:**
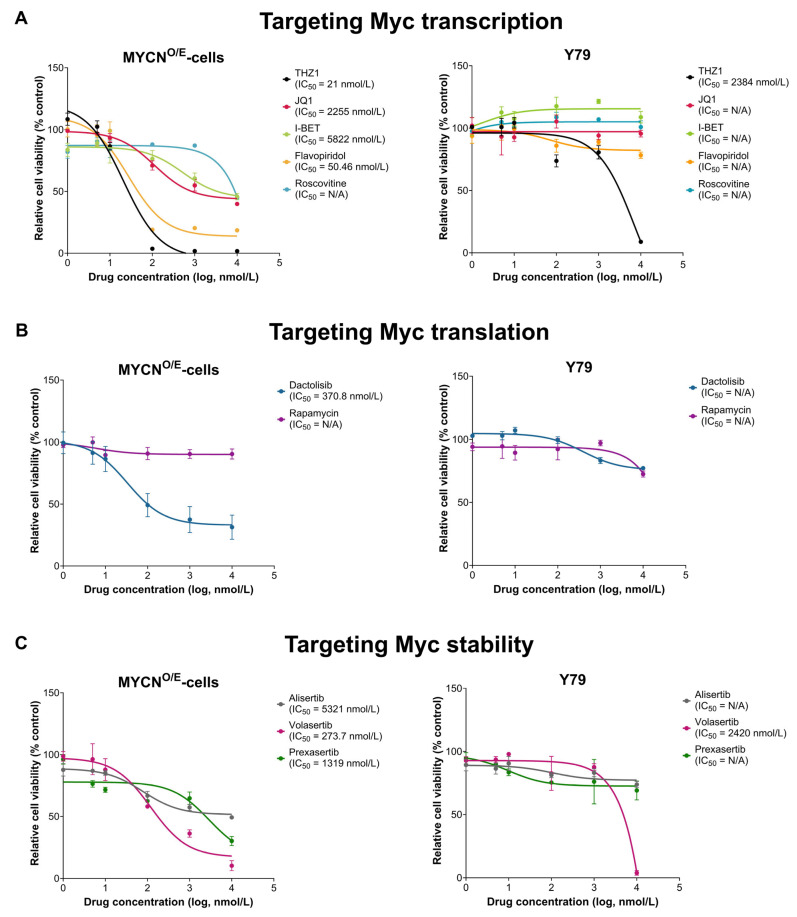
Selective Sensitivity of MYCN-Overexpressing Retinoblastoma Cells to MYC-Targeted Agents. Dose–response curves for MYCN^O/E^-cells and RB1-deficient Y79 cells following 48 h treatment with various small-molecule inhibitors. Data represent mean ± SEM (*n* = 3), with IC_50_ values indicated where applicable (N/A: not applicable due to insufficient efficacy). (**A**) Inhibitors targeting MYC transcription. MYCN^O/E^-cells exhibited marked sensitivity to the CDK inhibitors THZ1 (IC_50_ = 21 nM) and Flavopiridol (IC_50_ = 50.46 nM), whereas Y79 cells were largely resistant. (**B**) Inhibitors targeting MYC translation. The dual PI3K/mTOR inhibitor Dactolisib showed moderate efficacy against MYCN^O/E^-cells (IC_50_ = 370.8 nM), whereas the selective mTORC1 inhibitor Rapamycin was ineffective. In contrast, both inhibitors demonstrated negligible efficacy in Y79 cells (IC_50_ = N/A). (**C**) Inhibitors targeting MYC stability. The PLK1 inhibitor Volasertib demonstrated potency in the MYCN-driven subtype, effectively inhibiting MYCN^O/E^-cell viability (IC_50_ = 273.7 nM) with minimal impact on Y79 cells.

MYCN^O/E^-cells exhibited markedly increased sensitivity to specific MYC-targeted agents compared to Y79 cells (Figure 4). Among transcriptional inhibitors, the CDK7-selective inhibitor THZ1 demonstrated the most pronounced potency against MYCN^O/E^-cells (IC_50_ = 21 nM), whereas Y79 cells showed markedly lower sensitivity (IC_50_ = 2384 nM). Similarly, the broad-spectrum CDK inhibitor Flavopiridol displayed significant efficacy in MYCN^O/E^-cells (IC_50_ = 50.46 nM), while Y79 cells remained largely resistant, without a definable IC_50_. In contrast, other transcriptional inhibitors (JQ1, I-BET, Roscovitine) exhibited limited efficacy across both cell lines.

Within the translational inhibition group, the dual PI3K/AKT/mTOR inhibitor Dactolisib displayed moderate effectiveness in MYCN^O/E^-cells (IC_50_ = 370.8 nM), whereas Rapamycin, a selective mTORC1 inhibitor, showed negligible efficacy in both MYCN^O/E^-cells and Y79 cells. Regarding the modulation of MYC protein stability, Volasertib, a PLK1 inhibitor, significantly reduced the viability of MYCN^O/E^-cells (IC_50_ = 273.7 nM), whereas Y79 cells showed considerably reduced sensitivity (IC_50_ = 2420 nM). Other translation-targeting agents such as Alisertib and Prexasertib showed modest activity (IC_50_ values > 1000 nM), indicating limited therapeutic potential in both cell lines.

Collectively, these findings highlight a distinct therapeutic sensitivity profile of MYCN-overexpressing retinoblastoma cells, particularly to transcriptional inhibitors (THZ1, Flavopiridol) and the PLK1 inhibitor Volasertib. This selective sensitivity likely reflects a heightened dependency of these cells on MYC-driven signaling (‘oncogene addiction’), emphasizing the vulnerability of retinoblastoma cells primarily driven by MYCN amplification. In contrast, Y79 cells, characterized primarily by RB1 deficiency and broader genomic alterations, appear less dependent on MYC signaling, possibly due to compensatory activation of alternative survival pathways. These differential drug sensitivities underscore the importance of tailoring therapeutic strategies toward specific oncogenic dependencies in MYCN-driven retinoblastoma.

## 3. Discussion

In this study, we successfully established a novel human retinal organoid model of MYCN-driven retinoblastoma, demonstrating peak susceptibility to MYCN-mediated tumorigenesis within the developmental window of 70–120 days. Immunohistochemical analyses revealed that neoplastic cells emerging during this critical period exhibit robust proliferation (Ki-67 positive), strong expression of the retinal progenitor marker SOX2, and notably lack expression of the cone photoreceptor differentiation marker CRX.

A central outcome of our study is the refinement of the cellular origin for MYCN-amplified retinoblastoma. Previous seminal studies, primarily utilizing avian and organoid models, identified immature cone photoreceptor and horizontal cell progenitors as predominant originating cells [22]. Our results expand upon this by identifying a more primitive, SOX2-positive retinal progenitor, not yet committed to the photoreceptor lineage, as an additional susceptible population. Importantly, our findings do not necessarily contradict prior studies but rather suggest two complementary scenarios: MYCN may transform retinal progenitors at an earlier, undifferentiated stage than previously recognized [23], or it may induce dedifferentiation [24], causing photoreceptor-committed cells to revert to a progenitor-like state. Transcriptomic analyses demonstrate coordinated suppression of photoreceptor genes [24] and enhanced expression of neural and retinal ganglion cell progenitor markers [25], consistent with MYCN-driven lineage plasticity [25,26] and could underlie the aggressive phenotype observed [1,4], supporting either of these scenarios. Previous patient-derived and experimental studies predominantly implicated immature cone precursors as the cellular origin of MYCN-amplified retinoblastomas [23], often reporting concurrent expression of photoreceptor markers (CRX, ARR3) and neuronal markers (synaptophysin) [27], indicative of dedifferentiated or partially differentiated states. The differences between these studies and our findings reflect the inherent diversity and complexity of retinoblastoma cellular origins, suggesting that MYCN-driven retinoblastomas may originate from multiple retinal progenitor or precursor populations. Robust SOX2 expression is frequently observed in clinically aggressive, poorly differentiated retinoblastomas [28,29], correlating with invasiveness and stemness [30], further validating the primitive progenitor phenotype identified in our model.

The maximal tumor formation observed within the 70–120 day window likely reflects unique developmental characteristics of retinal progenitor populations during this specific period [31]. This transitional developmental stage is characterized by heightened proliferative activity and increased cellular plasticity, conditions highly favorable for oncogenic transformation. Retinal progenitor cells at this intermediate stage possess substantial proliferative capacity and increased susceptibility to genetic and epigenetic disruptions [32,33], facilitating the emergence of aggressive retinoblastoma.

In the early stages following MYCN overexpression, we observed a marked initial decline in GFP-positive cells, which was subsequently followed by the selective emergence and expansion of resistant tumor cell populations. This pattern may reflect an oncogenic selection process, wherein MYCN-overexpressing retinal cells undergo heightened oncogenic stress that can trigger apoptosis, while a minority of cells either acquire or inherently possess resistance mechanisms, allowing them to evade programmed cell death. Resistance to apoptosis is widely recognized as a fundamental hallmark of tumorigenesis across diverse cancer types [34,35], enabling genetically compromised cells to survive under otherwise lethal oncogenic stress. Supporting this interpretation, recent studies using MYCN-driven retinoblastoma models [22] have demonstrated that certain retinal progenitor lineages (cone and horizontal progenitors) display relative resistance to apoptosis, thereby facilitating MYCN-mediated tumorigenesis. Although our present data do not directly document apoptotic events, this explanation remains biologically plausible, and further experimental studies will be necessary to clarify the contribution of apoptosis resistance to the initiation and progression of MYCN-driven retinoblastoma.

In addition to refining the cell of origin, our transcriptomic analysis provided important insights into the molecular machinery driving this transformation. A key finding from our GSEA is the consistent activation of the mTORC1 signaling pathway in both our MYCN^O/E^-RBO model and clinical MYCN-amplified patient tumors. This is mechanistically significant, as mTORC1 is a central regulator of the anabolic metabolism required for cell growth [36], and MYCN is known to hijack this pathway to fuel the immense biosynthetic demands of rapid proliferation [2,37]. The convergence on this single pathway provides a powerful molecular explanation for the aggressive, hyper-proliferative phenotype of these tumors. This co-dependency also creates a clear therapeutic vulnerability, which we confirmed through pharmacological screening: the dual PI3K/mTOR inhibitor Dactolisib showed significant efficacy against our MYCN^O/E^-cells. This finding not only validates the clinical relevance of our model but also establishes the mTORC1 pathway as a rational and actionable therapeutic target for this aggressive retinoblastoma subtype.

Our pharmacological analyses highlight subtype-specific therapeutic vulnerabilities in retinoblastoma, underscoring that the tumor’s genetic background and primary oncogenic drivers profoundly influence therapeutic sensitivity [27,38,39]. MYCN-overexpressing retinoblastoma cells (MYCN^O/E^-cells) demonstrated remarkable sensitivity to MYC-targeting therapies, particularly transcriptional inhibitors such as THZ1 (a selective CDK7 inhibitor) and Flavopiridol (a broader-spectrum CDK inhibitor) [40,41]. The pronounced efficacy of THZ1 aligns well with CDK7’s established role in facilitating MYCN-dependent transcription, essential for sustained proliferation and survival [42]. Moreover, our screen identified significant sensitivity to the PLK1 inhibitor Volasertib [43], further reflecting heightened dependence on cell cycle and proliferative signaling pathways in MYCN-driven tumors [44].

Importantly, we observed a stark difference in drug sensitivity between MYCN^O/E^-cells and the classical RB1-deficient retinoblastoma cell line Y79, despite the latter carrying secondary MYCN amplification. The reduced sensitivity of Y79 cells suggests that RB1 loss, rather than MYCN amplification, serves as their primary oncogenic driver, activating multiple compensatory survival pathways [45,46] and reducing reliance on MYCN-driven signaling. In contrast, MYCN^O/E^-cells demonstrate a clear state of oncogene addiction [42], characterized by profound dependency on sustained MYCN transcriptional activity, rendering these cells particularly susceptible to targeted MYC inhibition. Collectively, our results not only validate MYCN as a critical therapeutic target but also underscores the necessity of a precision medicine approach tailored to the specific genetic context of this aggressive subtype.

Despite the significant strengths of our retinal organoid model in recapitulating the developmental and molecular features of MYCN-amplified retinoblastoma, several important limitations should be acknowledged. Organoid models inherently lack critical tumor microenvironment components such as immune interactions [47], vascular networks [48], and stromal influences [49], which are known to affect tumor progression and therapeutic responses in vivo. Furthermore, organoids derived from pluripotent stem cells can exhibit intrinsic variability in differentiation and maturity, potentially influencing reproducibility and interpretability of the results [50]. Lastly, the precise translation of organoid-based findings to clinical contexts remains challenging due to inherent differences between model systems and patient tumors [51]. Complementary studies employing additional preclinical models, including patient-derived xenografts and genetically engineered animal models, are necessary to fully validate and extend our findings toward meaningful clinical applications.

In conclusion, this study significantly enhances the understanding of MYCN-amplified retinoblastoma by establishing a robust, human-specific modeling platform—encompassing retinal organoids, derived cell lines, and orthotopic xenografts. Our findings identify a primitive, SOX2-positive retinal progenitor population and demonstrate a clear dependency on transcriptional and cell cycle machinery, providing a strong rationale for precision medicine approaches. However, the absence of critical microenvironmental factors represents an inherent limitation, necessitating complementary studies with animal and patient-derived models. Furthermore, organoid-derived tumors may lack spontaneous genetic heterogeneity present in patient tumors, potentially limiting insights into mechanisms of tumor evolution and resistance. Future research should prioritize validating these findings in more complex preclinical settings, exploring combinational therapies such as CDK7 inhibitors alongside other targeted agents, and translating these insights into clinical trials targeting this aggressive pediatric cancer subtype.

## 4. Materials and Methods

### 4.1. Cell Lines and Culture Conditions

Human embryonic stem cells (hESCs; H9, WiCell Research Institute, Madison, WI, USA) were maintained on Matrigel-coated plates (Corning, cat. no. 356231, Corning, NY, USA) in mTeSR™1 medium (STEMCELL Technologies, cat. no. 85850, Vancouver, BC, Canada). Culture medium was refreshed daily, and cells were passaged weekly using ReLeSR™ (STEMCELL Technologies, cat. no. 05872) with 10 μM Y-27632 (Sigma-Aldrich, cat. no. 688000, St. Louis, MO, USA). Retinoblastoma cell lines (Y79 and WERI-Rb1; ATCC, Manassas, VA, USA) were grown in suspension cultures in RPMI-1640 medium (Gibco™, cat. no. A1049101, Carlsbad, CA, USA) supplemented with 20% fetal bovine serum (FBS; Rd Tech, cat. no. A1500m, Namyangju, Republic of Korea) and 1% penicillin/streptomycin (Thermo Fisher Scientific, cat. no. 15140-122, Waltham, MA, USA). Human embryonic kidney (HEK293T; ATCC, Manassas, VA, USA) cells were cultured as adherent cells in DMEM (DMEM; Thermo Fisher Scientific, cat. no. 11995, Waltham, MA, USA) supplemented with 10% FBS and 1% penicillin/streptomycin. All cells were cultured at 37 °C in a humidified 5% CO_2_ incubator.

### 4.2. Retinal Organoid Differentiation

Human retinal organoids were differentiated from H9 cells following a previously described protocol with minor modifications [18]. Briefly, cells were dissociated using Accutase (BD Biosciences, cat. no. 561527, Franklin Lakes, NJ, USA) and seeded at a density of 3000 cells/well into 96-well ultra-low adhesion, round-bottom plates (Corning, cat. no. 7007, Corning, NY, USA). Cells aggregated over the following 24 h, then were cultured sequentially in BE6.2 medium supplemented with Wnt inhibitor (3 μM) (IWR1e: EMD Millipore, cat. no. 681669, Burlington, MA, USA) and Matrigel (1%) from days 1–6, BE6.2 with Matrigel (1%) on day 7, and plain BE6.2 from days 8–10. On days 10–12, retinal differentiation was induced using 100 nM Smoothened agonist (SAG; EMD Millipore, cat. no. 566660, Burlington, MA, USA). On day 10, aggregates were transferred to suspension cultures in untreated Petri dishes (Corning, cat. no. 3261, Corning, NY, USA), which prevents cell attachment and maintains the cultures in suspension. Retinal vesicles were manually isolated between days 10–14 using ultra-fine needles (BD Biosciences, Franklin Lakes, NJ, USA), then further cultured in long-term retina (LTR) medium containing 100 nM SAG. To enhance photoreceptor differentiation, organoids were treated with 1 μM all-trans retinoic acid (ATRA; Sigma-Aldrich, cat. no. R2625, St. Louis, MO, USA) from days 20–130 and 10 μM DAPT (γ-secretase inhibitor; EMD Millipore, cat. no. 565770, Burlington, MA, USA) from days 28–42. Media were refreshed every other day throughout differentiation.

### 4.3. Lentivirus Production and Transduction

HEK293T cells were transiently transfected with Lenti-MYCN-GFP (OriGene, cat. no. RC201241L4, Rockville, MD, USA), psPAX2 (Addgene, cat. no. 12260, Watertown, MA, USA), and pMD2.G (Addgene, cat. no. 12259) at a 4:3:1 ratio using Lipofectamine 2000 (Thermo Fisher Scientific, cat. no. 11668-019, Waltham, MA, USA). Lentiviral particles were harvested 48 and 72 h post-transfection, filtered (0.45 μm) (Millipore, cat. no. SLHVR33RS, Burlington, MA, USA), and concentrated by Vivaspin 20 (Sartorius, cat. no. VS2042, Göttingen, Germany) and ultracentrifugation (24,000 rpm, 2 h, 4 °C) in a SW28 rotor (Beckman Coulter, cat. no. 342207, Brea, CA, USA). Virus pellets were re-suspended in PBS and stored at −80 °C. Viral titers were quantified using a Lenti-X qRT-PCR Titration Kit (TaKaRa, cat. no. 631235, Shiga, Japan) following RNA extraction (TaKaRa, cat. no. 740956).

For lentiviral transduction, retinal organoids (8 per group) were incubated with lentivirus (4 × 109 viral genome copies/organoid) in LTR medium containing 8 μg/mL polybrene (Sigma-Aldrich, St. Louis, MO, USA) for 2 h, followed by culture for an additional 48 h. Organoids were then rinsed and transferred to ultra-low attachment plates for further culture.

### 4.4. Establishment of MYCN-Overexpressing Cell Lines (MYCN^O/E^-Cells)

To establish stable MYCN-overexpressing cell lines, GFP-positive tumor regions from MYCN-overexpressing retinal organoids (MYCN^O/E^-RBOs) were manually dissected and cultured in LTR medium on Matrigel-coated dishes. After approximately one week, cells were dissociated into single-cell suspensions using Accutase, seeded at a density of 1 × 10^5^ cells per well into ultra-low attachment plates, and grown as suspension cultures to generate tumor spheres.

### 4.5. Subretinal Xenograft Assays

All animal experiments were approved by the Institutional Animal Care and Use Committee (IACUC) at Yonsei University College of Medicine (Approval No: 2017-0239), following ARVO guidelines. 6-week-old NOD-SCID mice (Jackson Laboratory, strain code 001303, Bar Harbor, ME, USA) were anesthetized and dilated, after which MYCN^O/E^- cells (2 × 10^4^ cells in 2 μL PBS) were injected into the subretinal space using a 33-gauge sub-microliter injection system (NanoFil, World Precision Instruments, cat. no. UMP3-NF, Sarasota, FL, USA). Mice were euthanized 3–6 months post-injection when tumor formation was macroscopically evident, and eyes were harvested for histological analyses.

### 4.6. Histology and Immunostaining

Retinal organoids were fixed in 1% PFA (Biosesang, cat. no. P2031, Seongnam, Republic of Korea), cryoprotected in sucrose, and embedded in OCT compound (Sakura Finetek, cat. no. 4583, Torrance, CA, USA). Cryosections (7 µm) were permeabilized with 0.5% Triton X-100 (Sigma-Aldrich, cat. no. X100, St. Louis, MO, USA), blocked with 4% BSA (BSA; Sigma-Aldrich, cat. no. 821006), and incubated with primary antibodies overnight, followed by incubation with corresponding fluorophore-conjugated secondary antibodies. Nuclei were counterstained with DAPI (Vector Laboratories, cat. no. H-1200, Burlingame, CA, USA).

Enucleated eyes were fixed in 4% paraformaldehyde (PFA), embedded in paraffin, sectioned at 5 µm, and stained with hematoxylin and eosin (H&E). For immunohistochemistry (IHC), deparaffinized sections underwent heat-induced antigen retrieval in citrate buffer (pH 6.0) and were incubated with primary antibodies overnight at 4 °C, followed by detection with a DAB-based system.

Quantitative analysis of spatial colocalization between MYCN-GFP and specific cellular markers (Ki-67, SOX2, CRX) was performed using Pearson’s correlation coefficient (PCC). Immunofluorescent images of cryosectioned retinal organoids were acquired under identical microscopy settings and analyzed with the Coloc 2 plugin in Fiji-ImageJ software (version 1.54p) [52]. PCC values range from +1 (perfect colocalization) to −1 (perfect inverse correlation), with values close to zero indicating no correlation. PCC values greater than 0.5 were considered indicative of substantial colocalization. For each marker, analyses were conducted on the entire sectioned area of each organoid.

All images were acquired using an Olympus IX73 fluorescence microscope (Olympus Corporation, Tokyo, Japan). The primary antibodies used were mouse anti-CHX10 (1:200, Santa Cruz Biotechnology, cat. no. sc-365519, Dallas, TX, USA), mouse anti-CRX (1:1000, Abnova, cat. no. H0001406-M02, Taipei, Taiwan), rabbit anti-Recoverin (1:1000, Millipore, cat. no. AB5585, Burlington, MA, USA), mouse anti-SNCG (1:500, Abnova, cat. no. H00006623-M01A, Taipei, Taiwan), rabbit anti-Ki-67 (1:500, Abcam, cat. no. ab15580, Cambridge, UK), mouse anti-RXRγ (1:300, Santa Cruz Biotechnology, cat. no. sc-514134, Dallas, TX, USA), mouse anti-CRALBP (1:500, Abcam, cat. no. ab15051, Cambridge, UK), mouse anti-OTX2 (1:50, Santa Cruz Biotechnology, cat. no. sc-514195, Dallas, TX, USA), mouse anti-Rhodopsin (1:1000, Sigma-Aldrich, cat. no. R5403, St. Louis, MO, USA), and rabbit anti-L/M-opsin (1:500, Millipore, cat. no. AB5405, Burlington, MA, USA).

### 4.7. WST-1 Cell Viability Assay

To assess cell viability and drug sensitivity, 4 × 10^4^ serum-starved cells (Y79 and MYCN^O/E^-cells) were plated in 96-well plates and treated with various concentrations of candidate compounds for 48 h. WST-1 reagent (Roche Diagnostics, cat. no. 5015944001, Indianapolis, IN, USA) was added, incubated for 2 h, and absorbance was measured at 440 nm using a microplate reader.

### 4.8. RNA Sequencing and Analysis

#### 4.8.1. Data Collection

Transduced retinal organoids were manually dissected into tumor and normal regions using BD ultra-fine insulin syringes under a stereomicroscope, followed by dissociation with Accutase at 37 °C for 30 min. Total RNA was extracted using the RNA Clean & Concentrator Kit (Zymo Research, Orange, CA, USA) following the manufacturer’s protocol. Sequencing libraries were prepared using the Illumina Stranded Total RNA Prep Kit with Ribo-zero Plus (Illumina, San Diego, CA, USA) and were sequenced as 74 bp paired-end reads on a NextSeq 550 platform (Illumina). Library preparation and sequencing were performed by the Yonsei Genomics Center. For comparative analysis, two publicly available RNA-seq datasets were retrieved: data for the Y79 cell line (*n* = 8) from the NCBI Sequence Read Archive (SRA: PRJNA715828), and data from patient-derived tumors representing both RB1-mutant and MYCN-amplified cases from the Gene Expression Omnibus (GEO: GSE59983, GSE58780).

#### 4.8.2. Data Pre-Processing Pipeline

Quality assessment of raw sequencing data was conducted using FastQC v0.11.9. Adapter sequences and low-quality reads were trimmed with Trimmomatic v0.40 [53] using parameters: ILLUMINACLIP:2:30:10 LEADING:20 TRAILING:20 MINLEN:20 CROP:72. Trimmed reads were aligned to the human reference genome (GRCh38) using STAR v2.7.3a [54] with a two-pass mapping strategy. Gene-level read counts were generated from BAM files using HTSeq v0.11.1 [55]. Batch effects and non-biological variability were corrected using the ComBat-seq function from the SVA R package (v3.35.2). Genes with extremely low expression (average read count < 1 per sample across all groups) were excluded. Normalization was performed via variance-stabilizing transformation (VST) using DESeq2 (v1.26.0) [56].

#### 4.8.3. Differential Expression and Functional Analysis

Principal component analysis (PCA) was performed on normalized expression data from the top 500 genes showing the highest biological variation, visualized using prcomp in base R and ggplot2 (v3.3.6). Differential gene expression analysis was conducted with DESeq2, defining significant DEGs using criteria of adjusted *p*-value < 0.05 and |log2 fold change| > 1. Volcano plots were generated, and significantly up- and downregulated genes were used for downstream analyses. Functional enrichment analysis was carried out using the Database for Annotation, Visualization, and Integrated Discovery (DAVID v6.8), with significantly enriched Gene Ontology (GO) terms identified at an FDR < 0.05 and visualized using ggplot2.

#### 4.8.4. Gene Set Enrichment Analysis (GSEA)

Genes identified from differential expression analyses were ranked by descending log2 fold change for GSEA. Enrichment analysis was performed and visualized using the clusterProfiler R package (v3.14.3) [57] and gene sets (“c2.cp.kegg.v7.5.1.entrez.gmt”, “c5.go.bp.v.7.5.1.entrez.gmt”, “h.all.v.7.5.1.entrez.gmt”) obtained from the Molecular Signatures Database (MSigDB; http://software.broadinstitute.org/gsea/msigdb/index.jsp, accessed on 28 September 2022) using the msigdbr package (v7.5.1). Significance was assessed with 10,000 permutations, and results were considered statistically significant at *p* < 0.05.

### 4.9. Statistical Analysis

All quantitative data are presented as mean ± standard error of the mean (SEM) from at least three independent experiments. Statistical significance was determined using an unpaired two-tailed Student’s *t*-test for two-group comparisons or a one-way ANOVA with Tukey’s post hoc test for multiple-group comparisons. For the analysis of categorical data, such as the incidence of tumor formation, a chi-squared (χ^2^) test was employed, with Bonferroni correction for post hoc pairwise comparisons. All analyses were conducted using GraphPad Prism 8.3, and a *p* value < 0.05 was considered statistically significant.

## Data Availability

The original contributions presented in this study are included in the article. Further inquiries can be directed to the corresponding author(s).

## Supplementary Materials

The following supporting information can be downloaded at: https://www.mdpi.com/article/10.3390/ijms26178675/s1.

## Abbreviations

The following abbreviations are used in this manuscript:

RB	Retinoblastoma
MYCN^O/E^-RBOs	MYCN-overexpressing RB organoids
MYCN^O/E^-cells	MYCN-overexpressing cells
nROs	Normal retinal organoids
